# Mr. Moffatt, on Animal Chemistry

**Published:** 1801-11

**Authors:** J. M. Moffatt

**Affiliations:** Trowbridge


### *To the Editors of the Medical and Phyfical Journal.*

Gentlemen,

If you think the following defultory remarks on Animal Chemiftry will be any ways interefting to the readers of the Medical Journal, your infertion of them in that work will oblige,

Your's, &c.

*Trowbridge, Aug.* 28, 1801. J. M. MOFFATT.

---

It is rather extraordinary that the curious difcoveries of Galvani and others, relative to animal electricity, have not led to fome more important information concerning the functions of the nervous fyftem. Perhaps this may be owing to the attention of phyfiologifts not having been properly directed towards the effects of the electric fluid on the animal fibre. The late experiments of Volta* and Cruikfhank,† fufficiently prove, in oppofition to the opinion of Humboldt and others, that the Galvanic influence muft be electricity, or a modification of it. The opinion that the electric fluid is that power whereby the functions of the nervous fyftem are performed, has had fome adherents, ever fince the difcovery of the electrical powers of the torpedo and gymnotus electricus. But there is a book, which was publifhed more than thirty years fince, and probably written long before its publication, in which the identity of the electric and nervous fluids is hinted at.‡ The fame opinion has alfo found an advocate in Mr. Purton, the author of an ingenious paper publifhed in your

---

* Vide Monthly Magazine, Vol. IX. p. 586.

† Idem. Vol. X. p. 50.

‡ Vid. Syftemi de la Nature, Part. prem. p. 125, note (36). This impious publication is faid to have been compofed, in part at leaft, by the celebrated French philofopher Diderot.

your Journal.* But none of thofe writers, nor any others that I have been able to confult, have attempted to explain the *modus operandi* of this wonderful fluid. I fhall not, at prefent, endeavour to fupply the deficiency; the following hints and quotations being rather defigned to draw the attention of your readers towards the fubject in queftion.

Electricity is well known to be a powerful chemical agent; may we not therefore conjecture that it operates on the fyftem, by producing thefe chemical changes, the *tout enfemble* of which conftitutes life? This idea doubtlefs leads to the introduction of what has been called the chemical pathology; by the opponents of which I fhall probably be accufed of turning the human frame into a laboratory; and be told, that becaufe living animal fubftances have a power of preferving themfelves from decompofition, in fituations where dead animal fubftances would foon undergo that procefs, life muft depend on fomething that is exempt from the influence of chemical affinity. In anfwer, we may obferve, that the difference in the effects of air, &c. on living and dead animal bodies, may as eafily be accounted for, by fuppofing that death is a deduction of fome kind of matter which entered into the compofition of the living body, as by admitting the exiftence of a vital principle, or fome other equally undefinable agent. It is, at prefent, pretty generally allowed, that *some* of the functions neceffary to life depend on chemical affinity. Few will deny this to be the cafe with refpiration and digeftion. Reafoning from analogy, we may refer mufcular motion and fecretion to the fame caufe. The following quotations will fhow that this opinion is not wholly fingular.

"Les nerfs n'exercent-ils pas un effet chimique † fur les vaiffeaux fécrétoires? La décifion de cette queftion eft liée à la folution du grande problême, qui confifte à favoir fi toutes les modifications chimiques ne fe réduiffent pas à des mouvements; idée dont *Sage* s'eft beaucoup rapproché dans fa chimie mécanique. La contraction des mufcles elle-même ne ferait-elle pas le réfultat d'un mélange chimique; & fous ce point de vue, le mouvement n'eft-il pas liée aux affinités, comme celles-ci font au mouvement?" ‡

"On ne peut guère concevoir que les nerfs agiffent fur les fibres mufculaires, fans qu'il arrive un changement chymique dans la nature d'un fluide qui feroit contenu dans les uns par l'acceffion

* Vide Medical and Phyfical Journal, Vol. IV. p. 334, et feq.

† Roil & Gauthier, de Irritabilitate, 1795, p. 4.

‡ Expériences fur le Galvanifme de F. A. Humboldt; Traduction de l'Allemand, par Jadelot, p. 303.

l'acceffion de celui qu'y tranfmettroient les autres, ni que les objets extérieurs agiffent fur les nerfs autrement qu'en produifant un changement du même genre."*

The idea ftarted at the clofe of the latter of thefe quotations, that external objects give rife to ideas, by producing chemical changes in the nerves of fenfation, appears to be very ingenious, and highly worthy of attention; but the intricacy of the fubject will probably prevent Cuvier's conjecture from being verified by experiments.

Neither Humboldt nor Cuvier feems to have had any precife ideas relative to the nature of the chemical changes which they fuppofed to take place, but merely to have offered their remarks as conjectures worthy of farther confideration. As fuch I fhall leave them, for the prefent, to your readers.

Before I conclude, however, I fhall take the liberty to add, that fome facts which have occurred to me, have induced me to form a Theory of Mufcular Motion as depending on certain chemical proceffes. If the refults of fome intended experiments fhould corroborate thofe already made, I may probably lay this theory before the public.

* Leçons d'Anatomie Comparée de G. Cuvier, Tom. I. p. 52.

